# Injury-related characteristics and quality-of-life among Iranian individuals with spinal cord injury

**Published:** 2015-07-06

**Authors:** Hadis Sabour, Zahra Soltani, Sahar Latifi, Abbas Norouzi-Javidan, Farid Arman, Seyed Hassan Emami-Razavi, Seyed Mohammad Ghodsi, Mohammad Reza Hadian

**Affiliations:** 1 Brain and Spinal Cord Injury Research Center, Neuroscience Institute, Tehran University of Medical Sciences, Tehran, Iran; 2 Department of Psychiatry, Kermanshah University of Medical Sciences, Kermanshah, Iran

**Keywords:** Quality-of-Life, Spinal Cord Injury, Health Survey, Iran

## Abstract

**Background:** Health-related quality-of-life (HR-QOL) may be affected by various factors including injury-related characteristics among individuals with spinal cord injury (SCI). However, the impact of the influence of these variables has not yet been fully described in Iranian population. Here, we assessed the relationships between injury-related characteristics and HR-QOL among Iranian people with SCI.

**Methods: **HR-QOL was assessed using short-form health survey (SF-36). Referred patients to Brain and Spinal Injury Research Center between 2010 and 2012 were invited to participate in this investigation. Injury-related characteristics including injury level and completeness, time since injury, plegia type, and American Spinal Injury Association (ASIA) Impairment Scale were evaluated.

**Results: **Total of 104 patients (85 men and 19 women) entered the study. The majority of patients had a complete injury (77.9%). The most frequent ASIA score was A (75%), and the most common level of injury was at thoracic sections (61.5%). Lower injury levels were associated with higher scores in physical component summary (P = 0.040), mental component summary (P = 0.010) and subsequently total score (P = 0.006). Mean age and time since injury were 52.58 ± 12.69 and 10.88 ± 16.68 years, respectively, and were not related with HR-QOL (P = 0.70 and 0.220, respectively). There was no difference in terms of HR-QOL between patients with complete and incomplete injury. Paraplegic individuals had significantly higher scores in the domain of physical functioning compared to patients with tetraplegia (P = 0.007).

**Conclusion:** lower injury level is a significant predictor of better QOL among individuals with SCI whereas other injury-related characteristics including completeness, time since injury and plegia type may not influence HR-QOL.

## Introduction

Spinal cord injury (SCI) influences the life of affected individuals due to sensory and motor impairments along with increased risk of related secondary complications.^1^^-^^3^ By considering the increased incidence of SCI in developing countries,^4^ implementation of strategies to improve health-related quality-of-life (HR-QOL) among these people is essential.^5^ People with SCI tend to have lower level of physical, mental and social health and they also report lower level of well-being feeling.^6^^,^^7^ Many investigations have tried to identify determinants of quality-of-life (QOL) among people with SCI.^8^^,^^9^ Improving QOL is a major clinical goal and has become a key outcome measure in this population.^10^

HR-QOL presents self-perceived health status. HR-QOL contains two main domains: the physical and the mental.^11^^,^^12^ HR-QOL is dependent to many factors including self-esteem,^13^ marital status,^14^ post injury duration^15^ and injury level.^16^ Since both injury-related characteristics and environmental conditions can affect HR-QOL, levels of QOL may vary among people with SCI in different countries. However, many studies in different nations such as USA,^17^ Norway,^18^ Canada,^19^ and Sweden^20^ have shown lower levels of QOL in comparison with the general population. To our knowledge, limited investigations have assessed HR-QOL, and its related factors among Iranian individuals with SCI and most of these studies have focused on evaluating QOL in veterans.^16^^,^^21^^,^^22^ Here we tried to assess HR-QOL and its related variables among the Iranian population with SCI.

The aim of this study was to evaluate injury-related characteristics including injury level, completeness, time since injury and American Spinal Injury Association (ASIA) score on HR-QOL assessed by using a 36-item short-form (SF-36). SF-36 is a validated standard tool for assessment of QOL, and the Persian version of this measure has approved validity and reliability.^23^

## Materials and Methods

This is a cross-sectional investigation to evaluate HR-QOL in Iranian people with SCI. Participants were individuals with SCI, who were referred to Brain and Spinal Injury Research Center between November 2010 to April 2012. Exclusion criteria were: pregnancy, lactation, amputation, and non-traumatic SCI etiology. Patients with history of diabetes, cancer, endocrinology disease, acute infection, use of special medications such as glucocorticoid, hormones, thyroid hormones, anticonvulsive agents, heparin, aluminum-containing antacids, lithium, omega-3 fatty acids or other nutrients supplements, and smoking or alcohol consumption were also excluded. Patients with a history of addiction to illegal drugs were excluded as well. Written consent was obtained from each participant before enrollment. The study was approved by the ethics committee of Tehran University of Medical Sciences, Iran.

Patients’ age, gender, and time since injury were asked directly during interviews and were indexed in pre-prepared forms. Completeness of injury was defined as complete (no preserved sensory or motor function) or incomplete (variable motor function preserved below the neurological level of injury).^24^ Level of injury was assessed with clinical examinations and magnetic resonance Images and was confirmed by a neurologist. Classification of participants according to ASIA Impairment Scale was as follows: ASIA-A indicates complete injury with no preserved motor or sensory function below the neurological level. ASIA-B describes incomplete injury in which only sensory function is preserved below the neurological level. ASIA-C illustrates preserved motor function in which more than half of key muscles below the neurological level have a muscle grade < 3. ASIA-D indicates preserved motor function in which at least half of key muscles below the neurological level have a muscle grade of 3 or more. Only ASIA-A represents complete injury.^25^^,^^26^

HR-QOL was assessed using the SF-36 questionnaire. This instrument is a standard measurement tool for assessment of QOL and has been used for a long time among people with SCI. The psychometric properties of the Iranian version of the SF-36 questionnaire along with its validity and reliability are well-documented.^23^ This measurement tool includes 36 items which assess QOL in eight domains: physical functioning (PF), role limitation due to physical problems (RP), bodily pain (BP), general health perceptions (GH), vitality (VT), social functioning (SF), role limitation due to emotional problems (RE), and mental health (MH). These scales provide two component summary scores: physical component summary (PCS) and mental component summary (MCS). Scores range from 0 to 100, and higher scores are representative of better QOL.^27^^,^^28^ PCS includes domains of PF, RP, BP, and GH. MCS includes domains of VT, SF, role limitation due to RE, and MH.

All statistical analyses were performed using SPSS for Windows (version 21.0, SPSS Inc., Chicago, IL, USA). The chi-square test (Fisher’s exact test) was used to compare categorical variables in the univariate analysis. The comparison of SF-36 scores between groups was performed using one-way analysis of variance. Pearson correlation analysis was used to evaluate the relationship between continuous variables. Descriptive analysis with an expression of frequency and percentages for categorical variables and mean ± standard deviation (SD) for continuous values was presented. Age, time since injury, injury level, and completeness, ASIA score and plegia type (tetraplegia vs. paraplegia) were considered as independent variables. P < 0.050 was considered to be statistically significant.

## Results

Eighty-five men and 19 women with SCI participated in this study. The majority of patients were men (81.7%). Mean age was 51.86 ± 13.44 years in male participants and 56.05 ± 7.89 years in females which showed no significant difference between genders (P = 0.180). Seventy-eight (75.0%) had a complete injury and subsequently, the most common ASIA score was A (75.0%). The majority of participants were paraplegic (87.5%). The most frequent injury level was thoracic (61.5%) whereas 21 patients (20.2%) had an injury at the lumbar level, and only 19 subjects (18.3%) had an injury at the cervical level. Table 1 shows the baseline demographic characteristics among participants.

**Figure 1:**
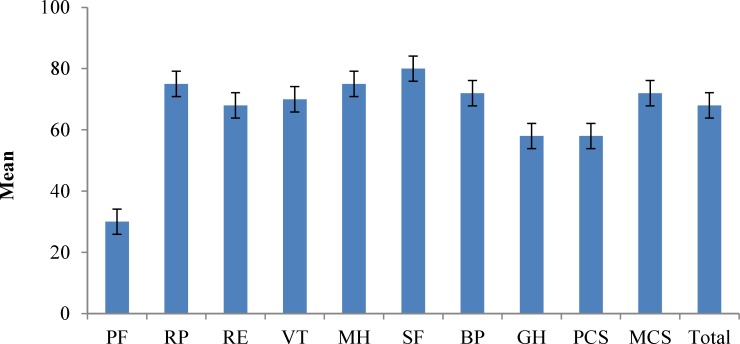
The obtained mean scores in domains of Short-Form-36 questionnaire

PF: Physical functioning; RP: Role limitation due to physical problems; RE: Role limitation due to emotional problems; VT: Vitality; MH: Mental health; SF Social functioning; BP: Bodily pail; GH: General health; PCS: physical component summary; MCS: mental component summary

Figure 1 illustrates the obtained mean scores in domains of the SF-36 questionnaire. Females had significantly higher scores in BP domain (P = 0.018). However the PCS, MCS, and the total score did not differ between men and women. Injury level was a determinant of HR-QOL. Scores in PF and VT were significantly higher among patients with injury at lumbar level (P < 0.0001 and 0.020, respectively) (Tables 2 and 3). PCS (P = 0.040), MCS (P = 0.010), and total scores (P = 0.006) were higher in patients with injury at lumbar level. However, completeness of injury was not associated with better HR-QOL. The mean total scores were 66.66 ± 14.9 and 61.20 ± 17.21 in patients with complete and incomplete injury, respectively (P = 0.18). On the other hand, ASIA-C was associated with lower total score. Mean total scores in ASIA-A, B, C, and D were 67.22 ± 14.3, 57.87 ± 18.4, 47.55 ± 16.9, and 69.41 ± 14.3, respectively (P: 0.04). However, there are some concerns about the reliability of this outcome since there were only 4 patients with ASIA-C. Moreover, patients with ASIA-D showed higher scores in VT (P = 0.020), BP (P = 0.001), and SF (P = 0.030) domains. Further analysis with grouping patients into two groups of paraplegics and tetraplegics revealed no association between type of plegia and scores of the SF-36 questionnaire (P = 0.34). However, paraplegic individuals had significantly better scores in the domain of physical functioning (P = 0.007) (Table 3).

Correlation analysis detected no significant association between age and scores of PCS (P = 0.25) and MCS (P = 0.55) and the effect of age on total score of SF-36 questionnaire was also insignificant (P = 0.70). Mean time since injury was 9.26 ± 6.32. Time since injury had no influence on HR-QOL and the relationships between time since injury and PCS (P = 0.430), MCS (P = 0.180), and total scores (P = 0.220).

**Table 1:** Baseline characteristics in participants with spinal cord injury

**Category**	**Frequency (%)**	**Mean ± SD**
Gender		
Male	85 (81.7)	-
Female	19 (18.3)	-
Age (year)		52.58 ± 12.69
Completeness		
Complete	78 (75.0)	-
Incomplete	26 (25.0)	-
ASIA score		
A	78 (75.0)	-
B	12 (11.5)	-
C	4 (3.8)	-
D	10 (9.6)	-
Plegia		
Paraplegia	91 (87.5)	-
Tetraplegia	13 (12.5)	-
Time since injury (years)	-	9.26 ± 6.32

ASIA: American Spinal Injury Association; SD: Standard deviation

**Table 2:** Scores of short-form-36 (SF-36) questionnaire domains in patients with spinal cord injury classified according to injury characteristics

**Category**	**PF**	**RP**	**BP**	**GH**	**VT**	**SF**	**RE**	**MH**	**PCS**	**MCS**	**Total**
Gender											
Male	29.35 (22.6)	71.64 (39.8)	75.12 (24.7)	58.25 (22.2)	70.0 (15.7)	82.07 (22.5)	67.48 (42.1)	76.0 (17.2)	58.18 (15.8)	73.9 (18.5)	66.40 (14.7)
Female	18.82(12.1)	69.44 (40.7)	58.75 (25.6)	54.11 (27.1)	61.76 (18.3)	69.85 (23.4)	62.22 (48.5)	68.70 (19.7)	54.19 (19.6)	66.65 (21.7)	60.14 (18.8)
Injury level											
Cervical	11.57 (15.0)	71.25 (40.8)	68.25 (31.3)	51.31 (26.3)	65.25 (14.1)	74.40 (25.4)	54.38 (48.7)	71.57 (15.6)	50.36 (18.8)	65.53 (19.8)	58.30 (16.9)
Thoracic	29.47 (20.2)	67.74 (40.8)	72.2 (23.2)	59.58 (22.2)	70.60 (17.5)	80.32 (22.7)	68.36 (41.7)	74.98 (19.8)	57.63 (13.7)	74.36 (19.3)	66.31 (14.0)
Lumbar	37.77 (23.1)	83.34 (34.3)	78.05 (26.2)	57.22 (22.4)	85.29 (14.8)	85.41 (20.6)	74.07 (40.5)	77.11 (12.5)	74.09 (19.4)	84.95 (17.2)	89.60 (16.9)
Completeness of injury											
Complete	26.38 (18.3)	72.11 (39.3)	73.83 (23.2)	59.07 (22.4)	70.34 (16.2)	80.68 (22.9)	69.77 (41.8)	75.14 (18.3)	58.04 (14.5)	74.48 (18.6)	66.66 (14.9)
Incomplete	30.90 (29.8)	68.18 (42.4)	67.72 (32.2)	51.09 (25.1)	62.50 (16.1)	77.71 (23.5)	55.55 (46.3)	73.14 (15.9)	55.87 (22.1)	66.53 (20.4)	61.20 (17.2)
ASIA score											
A	27.07 (18.8)	74.0 (38.6)	75.40 (22.4)	60.75 (22.1)	70.21 (16.8)	70.74 (23.2)	69.44 (42.1)	74.94 (18.7)	59.50 (13.8)	74.33 (19.1)	67.22 (14.3)
B	21.66 (11.5)	60.41 (45.8)	47.29 (31.2)	54.58 (20.5)	63.33 (16.5)	82.29 (23.5)	52.77 (45.9)	80.66 (11.7)	45.98 (19.8)	69.76 (19.7)	57.87 (18.4)
C	22.50 (38.6)	67.50 (43.3)	64.37 (37.4)	35.0 (8.66)	67.50 (10.4)	88.87 (21.3)	58.33 (50.0)	69.33 (10.0)	35.20 (24.3)	59.90 (13.5)	47.55 (16.9)
D	31.87 (20.8)	77.78 (36.3)	85.56 (15.6)	42.77 (28.9)	85.78 (24.1)	90.01 (23.8)	66.67 (47.1)	66.80 (17.3)	68.30 (15.8)	68.32 (20.8)	69.41 (14.2)
Plegia											
Paraplegia	29.69 (21.3)	70.40 (39.9)	72.12 (24.6)	58.70 (21.8)	68.59 (16.8)	81.17 (21.9)	67.06 (42.5)	75.38 (17.4)	58.06 (15.9)	73.48 (18.9)	65.95 (15.3)
Tetraplegia	12.08 (16.3)	76.92 (40.1)	75.0 (32.5)	49.16 (30.4)	68.07 (14.3)	72.11 (28.9)	63.88 (48.1)	70.0 (18.3)	53.50 (20.4)	67.03 (20.9)	60.93 (17.5)

ASIA: American Spinal Injury Association; PF: Physical functioning; RP: Role limitation due to physical problems; BP: Bodily pain; GH: General health; VT: Vitality; SF: Social functioning; RE: Role limitation due to emotional problems; MH: Mental Health

**Table 3:** P values in the relationships between injury characteristics and health-related quality of life assessed by short-form-36 (SF-36) questionnaire

**Category**	**PF**	**RP**	**BP**	**GH**	**VT**	**SF**	**RE**	**MH**	**PCS**	**MCS**	**Total**
Gender	0.0670	0.83	0.018	0.50	0.610	0.05	0.66	0.12	0.400	0.180	0.170
Injury Level	< 0.0001**	0.35	0.490	0.40	0.021*	0.32	0.34	0.63	0.045*	0.011*	0.006**
Injury completeness	0.3900	0.68	0.320	0.20	0.760	0.59	0.18	0.65	0.600	0.100	0.180
ASIA score	0.1900	0.22	0.001**	0.05	0.027*	0.038*	0.64	0.32	0.140	0.013*	0.040*
Plegia	0.0070**	0.58	0.710	0.18	0.810	0.33	0.81	0.83	0.410	0.300	0.340

ASIA: American Spinal Injury Association; PF: Physical functioning; RP: Role limitation due to physical problems; BP: Bodily pain; GH: General Health; VT: Vitality; SF: Social functioning; RE: Role limitation due to emotional problems; MH: Mental health; PCS: Physical component summary; MCS: Metal Component Summary

* Statistically significant at the 0.05 level;

** Statistically significant at the 0.01 level

## Discussion

The findings of this study illustrate that level of the injury is the major determinant of QOL in patients with SCI. It is well-described that higher level of injury is associated with more severe muscle loss and decreased muscle strength and performance which may contribute to lower HR-QOL.^29^ Jain et al.^29^ demonstrated that higher injury level and complete injuries are associated with poorer QOL. Although our investigation has shown similar results on the effect of injury level, no relationship between injury completeness and HR-QOL could be detected in our study. In line with our results, several studies have illustrated the insignificant influence of injury completeness on QOL.^30^^,^^31^ Some investigations have described that complete motor lesions may lead to increased likelihood of occurrence of pressure ulcers and other related complications by limiting the patients to wheelchair^32^ which may contribute to poorer QOL in comparison with patients with incomplete injury.^33^ However, patients with incomplete injuries may be limited to wheelchairs as well, and thus completeness of injury may not be the single factor affecting QOL. Existence of various factors which influence QOL may play a role in these conflicting outcomes. However, it seems that completeness of injury is not a major determinant of QOL among individuals with SCI whereas the level of injury plays an important role in determining the level of QOL among these people.

No significant relationship could be found between age and HR-QOL, which contradicts with some of the previous investigations which had shown a negative effect of older ages on QOL.^19^^,^^29^ In line with our results, Cushman and Hassett^34^ and Barker et al.^35^ reported no association between age and QOL. Since QOL is affected by various factors such as educational level, employment status, income, social activities and familial support,^36^ the relationships between these variables may vary among nations due to existence of different environmental conditions. In fact, the association between age and HR-QOL can be affected due to the existence of these confounders, and it is emphasized to perform multivariate analysis with control for confounders in each population. In this regard, Ebrahimzadeh et al.^22^ showed that age was not related with HR-QOL in Iranian population with SCI, which approves our results.

This study shows no association between time since injury and HR-QOL as well. These results are in line with previous reports in Ebrahimzadeh et al.,^22^ Cushman and Hassett^34^ and Barker et al.^35^ studies. On the other hand, Geyh et al.^37^ demonstrated that shorter time since the injury is a significant predictor of lower QOL which contradicts with our results. According to Wijesuriya et al. study,^38^ shorter time since injury was significantly associated with higher levels of fatigue among individuals with SCI. It seems that the association between time since injury and QOL can be affected by other factors such as fatigue which may explain the significant contribution of shorter time since injury in lower QOL in Geyh et al.’s findings. More investigations with control for these confounders should be performed to understand the association between time since injury and QOL.^37^

Previously, Lin et al.^39^ reported that tetrapegics have poorer QOL in comparison with people with paraplegia. However our results difference HR-QOL between patients with tetraplegia vs. paraplegia which is in line with Lidal et al.^18^ and Ebrahimzadeh et al.^22^ studies. One probable reason, which has also been described by Ebrahimzadeh et al., may be the existence of accessible facilities and recreational programs for patients with tetraplegia which enables them to participate in social contributions and improves their degree of dependency.^22^ According to our study, paraplegic individuals had significantly better physical functioning compared with patients with tetraplegia. It seems that although recreational and rehabilitation programs may compensate the higher level of dependency in patients with tetraplegia to some extent, still paraplegic individuals have significantly better QOL in the domain of physical functioning.

Lidal et al.^18^ found no significant difference in the HR-QOL between patients with ASIA Impairment Scale A-C versus D-E. However, Kivisild et al.^40^ showed that ASIA scale can be a significant predictor of PF domain in the acute phase of the injury. In this study, people with ASIA-B showed higher scores in domains of BP, VT, and SF in comparison with ASIA-A. People with ASIA-A have a complete injury with no preserved sensory and motor functions whereas in ASIA-B, the sensory function is preserved to some extent. It seems that this preserved sensory function contribute to better QOL in patients with ASIA B in comparison with ASIA-A. However, a conflicting outcome which was detected in our analysis was the lower total scores of SF-36 questionnaire among patients with ASIA-C. It is noticeable that there may be some concerns about the reliability of analysis in patients with ASIA-C since only four patients with ASIA-C participated in our investigation. Altogether, it can be concluded from our results that ASIA-B is accompanied with better QOL in comparison with ASIA-A. However, further investigation with larger sample size may be required to clarify the association between ASIA impairment Scale and HR-QOL.

## Conclusion

This investigation shows that lower injury level is a significant predictor of better QOL among individuals with SCI whereas other injury-related characteristics including completeness, time since injury and plegia type may not influence HR-QOL. Age and gender were not determinants of QOL as well.

## Notes:

**How to cite this article: **Sabour H, Soltani Z, Latifi S, Norouzi-Javidan A, Arman F, Emami-Razavi SH, et al. Injury-related characteristics and quality-of-life among Iranian individuals with spinal cord injury. Iran J Neurol 2015; 14(3): 136-41.
